# High-intensity statin treatment is associated with reduced plaque structural stress and remodelling of artery geometry and plaque architecture

**DOI:** 10.1093/ehjopen/oeab039

**Published:** 2021-11-17

**Authors:** Sophie Z Gu, Charis Costopoulos, Yuan Huang, Christos Bourantas, Adam Woolf, Chang Sun, Zhongzhao Teng, Sylvain Losdat, Lorenz Räber, Habib Samady, Martin R Bennett

**Affiliations:** 1 Division of Cardiovascular Medicine, University of Cambridge, Level 6, ACCI, Addenbrooke’s Hospital, Hills Road, Cambridge CB2 0QQ, UK; 2 Department of Cardiology, Royal Papworth Hospital, Papworth Road, Cambridge CB2 0AY, UK; 3 Centre for Mathematical and Statistical Analysis of Multimodal Imaging, University of Cambridge, 20 Clarkson Road, Cambridge CB3 0EH, UK; 4 Department of Radiology, University of Cambridge, Hills Road, Addenbrooke’s Hospital, Cambridge CB2 0QQ, UK; 5 Institute of Cardiovascular Sciences, University College London, 62 Huntley Street, London WC1E 6DD, UK; 6 Department of Cardiology, Barts Heart Centre, West Smithfield, London EC1A 7BE, UK; 7 Department of Engineering, University of Cambridge, Trumpington Street, Cambridge CB2 1PZ, UK; 8 Institute of Social and Preventive Medicine and Clinical Trials Unit, University of Bern, Hochschulstrasse 6, Bern 3012, Switzerland; 9 Department of Cardiology, Bern University Hospital, Freiburgstrasse 18, 3010 Bern, Switzerland; 10 Division of Cardiology, Department of Medicine, Emory University School of Medicine, 201 Dowman Drive, Atlanta, GA 30322, USA

**Keywords:** Atherosclerosis, Plaque architecture, Plaque structural stress, Virtual-histology intravascular ultrasound

## Abstract

**Aims:**

Plaque structural stress (PSS) is a major cause of atherosclerotic plaque rupture and major adverse cardiovascular events (MACE). We examined the predictors of changes in peak and mean PSS (ΔPSS_peak_, ΔPSS_mean_) in three studies of patients receiving either standard medical or high-intensity statin (HIS) treatment.

**Methods and results:**

We examined changes in PSS, plaque size, and composition between 7348 co-registered baseline and follow-up virtual-histology intravascular ultrasound images in patients receiving standard medical treatment (controls, *n* = 18) or HIS (atorvastatin 80 mg, *n* = 20, or rosuvastatin 40 mg, *n* = 22). The relationship between changes in PSS_peak_ and plaque burden (PB) differed significantly between HIS and control groups (*P* < 0.001). Notably, PSS_peak_ increased significantly in control lesions with PB >60% (*P* = 0.04), but not with HIS treatment. However, ΔPSS_peak_ correlated poorly with changes in lumen and plaque area or PB, plaque composition, or lipid lowering. In contrast, ΔPSS_peak_ correlated significantly with changes in lumen curvature, irregularity, and roughness (*P* < 0.05), all of which were reduced in HIS patients. ΔPSS_mean_ correlated with changes in lumen area, PA, PB, and circumferential calcification, and was unchanged with either treatment.

**Conclusion:**

Our observational study shows that PSS_peak_ changes over time were associated with baseline disease severity and treatment. The PSS_peak_ increase seen in advanced lesions with standard treatment was associated with remodelling artery geometry and plaque architecture, but this was not seen after HIS treatment. Smoothing plaques by reducing plaque/lumen roughness, irregularity, and curvature represents a novel mechanism whereby HIS may reduce PSS and, thus may protect against plaque rupture and MACE.

## Graphical Abstract

**Graphical Abstract:**
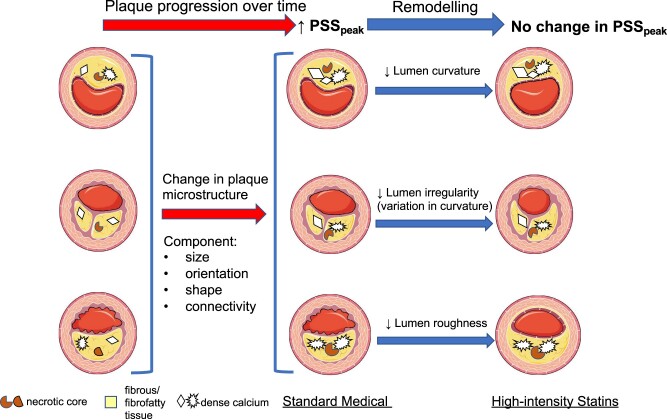
Plaques progress over time with standard medical treatment due to changes in plaque microstructure, resulting in increased peak PSS. High-intensity statins remodel the lumen/plaque interface, reducing lumen curvature, irregularity and roughness, and preventing the increase in peak PSS seen with standard medical treatment. Smoothing plaques and reducing lumen curvature represent novel mechanisms whereby high-intensity statins may protect against plaque rupture. PSS, plaque structural stress.

## Introduction

Despite current optimal medical and interventional management, patients with coronary artery disease (CAD) have significant risk of future major adverse cardiovascular events (MACE).^1^^,^^2^ In particular, patients presenting with acute coronary syndrome (ACS) demonstrate multiple vulnerable plaques and simultaneous plaque ruptures in non-culprit vessels,^3^ confirming the multifocal nature of unstable atherosclerosis, and prospective studies show that 50% of future MACE occur in non-culprit vessels.^4^^,^^5^ Lipid-lowering with statins and proprotein convertase subtilisin kexin type 9 (PCSK9) inhibitors reduce MACE by 25–40%,^1^^,^^2^^,^^6^ despite modest reductions in lumen stenosis^7^ and <1% reduction in whole-vessel percent atheroma volume (PAV),^8^^,^^9^ suggesting that these drugs may stabilize plaques, for example by increasing fibrous tissue (FT) and reducing necrotic core (NC). However, virtual-histology intravascular ultrasound (VH-IVUS) studies show only small or no change in FT or NC areas after statins^10^^,^^11^ or PCSK9 inhibitors,^12^ suggesting that changes in plaque composition alone do not fully explain their marked clinical benefit.

Coronary plaques undergo mechanical loading due to dynamic changes in blood pressure and flow,^13^ with rupture occurring if plaque structural stress (PSS) exceeds its mechanical strength. PSS can be calculated from arterial and plaque geometry, plaque composition, tissue material properties (defined from *ex vivo* tensile testing), and blood pressure. Maximal PSS (PSS_peak_) is increased at higher-risk plaques in ACS vs. stable angina patients,^14^ at rupture sites vs. stable lesions,^15^ and plaques associated with future MACE.^16^^,^^17^ Furthermore, increased baseline PSS is associated with changes to a more ‘vulnerable plaque’ phenotype over time.^18^ However, how PSS changes over time, the major predictors of these changes, and whether lipid-lowering affects PSS are unknown. We examined changes in PSS in three studies of patients receiving either standard medical treatment or high-intensity statins (HIS). We find that PSS increases in advanced lesions with standard medical but not HIS treatment, associated with remodelling artery geometry and plaque architecture.

## Methods

### Studies

Studies were conducted at Emory University Hospital, USA, and Bern University Hospital, Switzerland. All studies were approved by institutional review boards (ClinicalTrials.gov: NCT00576576, NCT01230892, and NCT00962416), and all patients provided informed consent and underwent protocol-driven baseline and follow-up angiography and VH-IVUS.

Control patients received standard medical treatment, which included aspirin, low-intensity statin, and a β-blocker for 12 m (*n* = 18). HIS patients received either atorvastatin 80 mg for 6 m (*n* = 20) or rosuvastatin 40 mg for 13 m (*n* = 22). Control patients presented with stable angina with an abnormal non-invasive stress test, or ACS with moderate but non-obstructive lesions [plaque burden (PB) ≥40%, <50% stenosis visually by angiography, or <70% stenosis with fractional flow reserve (FFR) >0.80].^19^ Atorvastatin-treated patients presented with either stable angina or ACS with moderate lesions, while rosuvastatin-treated patients presented with ST-segment elevation myocardial infarction, with study of moderate lesions in non-culprit vessels.^20^^,^^21^ We analysed only left anterior descending arteries as US studies included only these arteries.

### Virtual-histology intravascular ultrasound

Images were acquired with phased-array 20-MHz Eagle Eye catheters (Volcano Corp., Rancho Cordova, USA) using 0.5-mm/s automated motorized pullback. Radiofrequency data were captured on the R-wave using ECG-triggered acquisition. All images underwent quality control assessment by experienced investigators blinded to clinical data at Emory Cardiovascular Imaging and Biomechanical Core Laboratory (control and atorvastatin) or Cardialysis B.V., Rotterdam (rosuvastatin). Data were analysed offline using echoPlaque 4.0 (Indec Medical, San Jose, USA) and QIVUS software (Medis, Leiden, Netherlands). Lumen and external elastic membrane (EEM) areas, plaque area (PA, defined as plaque and media = EEM − lumen areas), PB (defined as 100% × PA/EEM area), and plaque composition [fibrofatty (FF), FT, NC, and dense calcium (DC) area, and percentage] were calculated between baseline and follow-up.

Baseline and follow-up VH-IVUS frames were co-registered longitudinally using anatomical landmarks (e.g. side branches, stenosis, calcification/large lipid cores). Frames were rotated using anatomical landmarks and lumen shape matching for circumferential alignment. Matching was confirmed by two analysts and showed good reproducibility (see Supplementary material online).

### Lumen analysis

We also calculated lumen aspect ratio (ratio between maximum/minimum diameter of ellipse) to measure lumen eccentricity, lumen curvature (computed using the radius of the circle determined by the point of interest and 2 adjacent points) to measure lumen angulation, lumen irregularity (variation in luminal angulation), and lumen roughness (lumen surface evenness in respect to curvature) (Supplemental material online, *Figure S1*).^22^

### Biomechanical analysis

Each vessel generated an average of 169 (136–212) (median, interquartile range) baseline and follow-up VH-IVUS frames (total = 10 517 frames). A total of 4933 frames with <30% stenosis or containing significant side branches or immediately adjacent to bifurcations were excluded from finite element analysis (FEA) due to violating the plane strain assumption for 2-dimensional (2D) solid modelling. Vessel geometry and plaque composition were extracted and 2D dynamic FEA simulations performed as described previously (Supplementary material online).^16^ Maximum principal stress in the peri-luminal region was used to indicate critical mechanical conditions within the structure.

### Statistical analysis

As each plaque had multiple VH-IVUS frames, linear mixed-effects (LME) models were used to account for hierarchical data structure and clustering in individual patients undergoing different treatments, and results presented as mean ± standard error (SE). All plaque anatomical, geometric, compositional or PSS measurements were analysed by LME on frame-based data in each patient unless otherwise indicated. Adjustment of *P* values for multiple comparisons was performed using the Bonferroni method. Potential confounding factors were included in multivariable regression analyses to assess robustness of the main study findings. Model diagnostics were performed by inspecting residual and Q–Q plots to test model assumptions. Outliers were removed using the median absolute deviation method (threshold 3.5). Association between continuous variables was assessed by Pearson’s correlation coefficient and linear regression. Regression slopes were compared using the analysis of covariance test. Statistical significance was indicated by two-tailed *P*-value <0.05. Statistical Package for the Social Sciences (SPSS, version 26.0; IBM, New York, USA) and R 4.0.3 (R Foundation for Statistical Computing) were used for all statistical analyses. The corresponding author had full access to all study data and takes responsibility for its integrity and data analysis.

## Results

### Study populations

**Figure 1:**
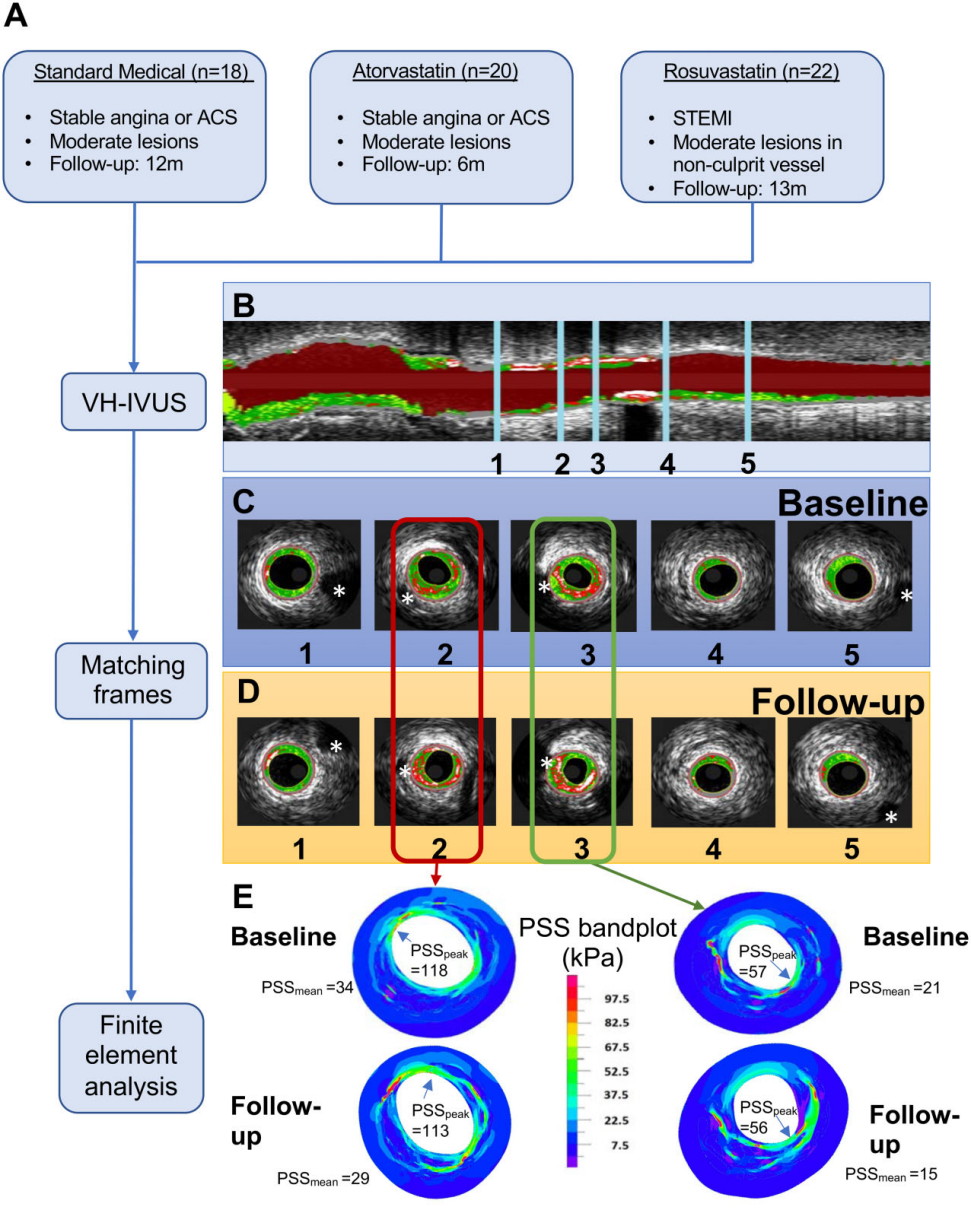
Study workflow and co-registration of baseline and follow-up virtual-histology intravascular ultrasound images and corresponding plaque structural stress band plots. (*A*) Flow chart of study workflow. (*B*) Longitudinal view of virtual-histology intravascular ultrasound pullback with 5 marked points. The segment of interest was defined using the most proximal and distal side branches (marked with *) seen at baseline (*C*) and follow-up (1, 5) (*D*). Other side branches (2, 3) or fiduciary marks were used to identify corresponding frames, and an interpolation technique applied to find corresponding frames in segments with no landmarks (4). (*E*) Examples of peak and mean plaque structural stress (PSS_peak_ and PSS_mean_) band plots for marked points 2 and 3 at baseline and follow-up. ACS, acute coronary syndrome; STEMI, ST-segment elevation myocardial infarction.

We examined PSS and plaque characteristics at baseline and follow-up in patients treated with either: (i) Standard medical therapy including low-intensity statins for 12 m (controls) or (ii) either 80 mg atorvastatin for 6 m or 40 mg rosuvastatin for 13 m (HIS) in three separate trials (*Figure 1*). Full trial details and patient demographics are shown in Supplementary material online, *Tables S1* and *S2*. Control and atorvastatin patients had similar baseline demographics, but more control patients had prior statin and nitrate use. The rosuvastatin group had more males and smokers, and fewer prior statin, anticoagulation or angina medications compared to controls. Low-density lipoprotein (LDL) levels increased slightly over time in control patients, most likely from patient discontinuation of standard treatment, but were reduced in atorvastatin and rosuvastatin patients; there was no difference in changes in high-density lipoprotein (HDL) levels or blood pressure reduction between groups. Baseline plaque characteristics were largely similar between control and atorvastatin patients, including VH-IVUS-determined plaque composition, but rosuvastatin patients had smaller EEM, lumen areas, and FT% and higher PB but not PA vs. controls (Supplementary material online, *Table S3*). We therefore co-registered images (*Figure 1*) and analysed changes between baseline and follow-up images for each treatment, with each patient acting as their own control, rather than direct comparisons between groups.

### Changes in PSS with treatment

**Figure 2:**
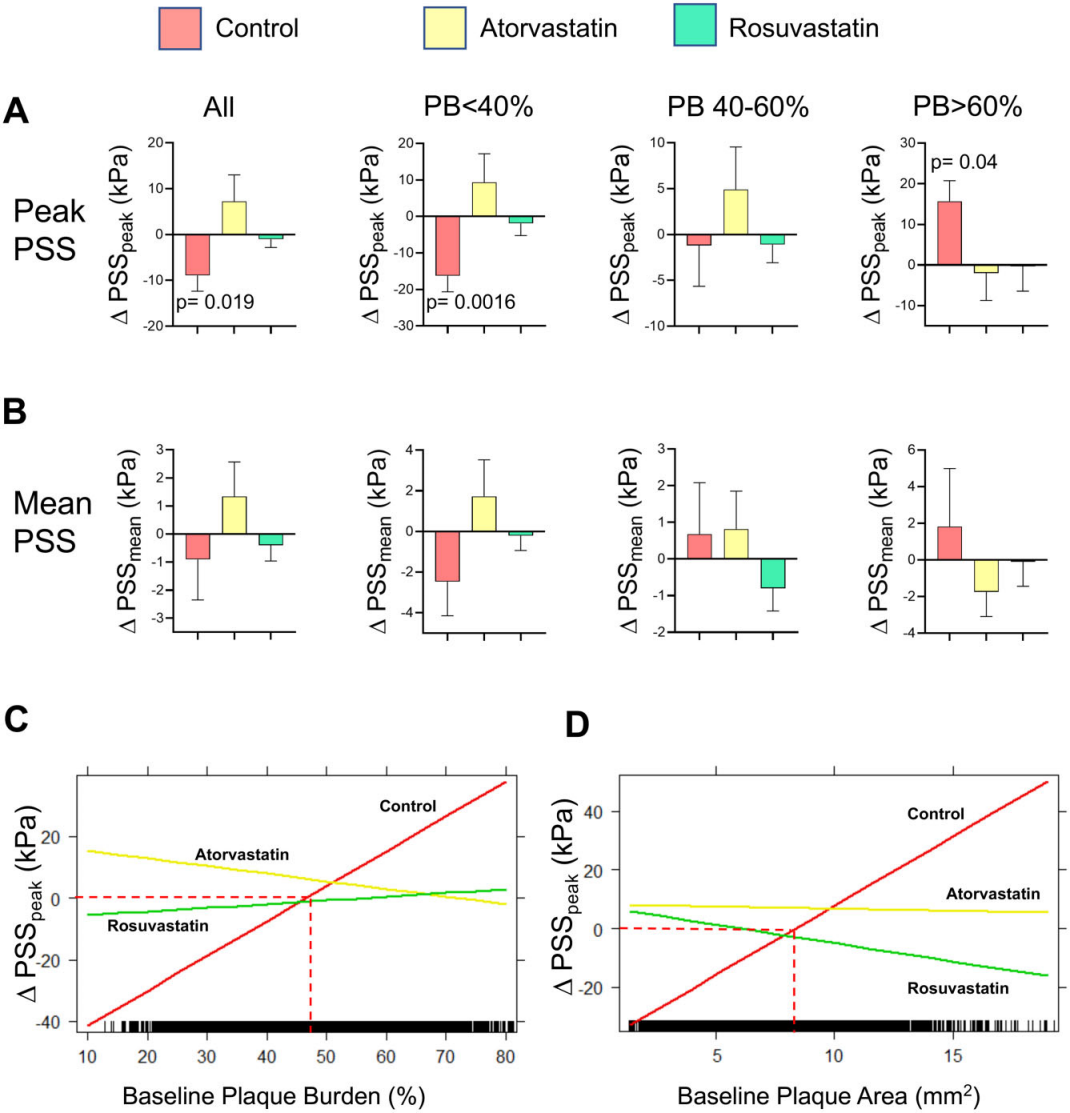
Changes in peak and mean plaque structural stress with baseline plaque burden after control or high-intensity statin treatment. Change in PSS_peak_ (*A*) or PSS_mean_ (*B*) in all plaques or with different plaque burden in control patients or treated with high-intensity statins. Data are mean (standard error), using mixed-effects models. Interaction plots of linear mixed-effects models showing significant interaction effect of treatment group and baseline plaque burden (*C*) or plaque area (*D*) on ΔPSS_peak_. PB, plaque burden; PSS, plaque structural stress.

Maximal PSS (PSS_peak_) and mean PSS (PSS_mean_) were calculated for each frame (total *n* = 7348 frames). PSS_peak_ was reduced overall in control patients, but this effect was due to small lesions (PB < 40%, *Figure 2A*) whose clinical significance is unclear, as PB is a major predictor of MACE in prospective VH-IVUS trials.^4^^,^^5^^,^^23^ In contrast, PSS_peak_ was unchanged in moderate lesions (PB 40–60%) in control patients but increased significantly in PB >60% lesions (15.6 ± 5.3 kPa, mean ± SE, *P* = 0.04). Atorvastatin and rosuvastatin patients showed no change in PSS_peak_ at any PB, and particularly no rise in PSS_peak_ in PB >60% lesions (*Figure 2A*). Analysis of 2 mm axial segments showed broadly similar findings (Supplementary material online, *Table S4*), while mean PSS was unchanged in control, atorvastatin, or rosuvastatin patients at any PB (*Figure 2B*).

Our findings suggest that the major effect of HIS on PSS_peak_ is on advanced lesions (PB >60%). We therefore used interaction plots of LME models to examine interaction effects of treatment group and baseline PB or PA on changes in PSS_peak._ There was a significant interaction between ΔPSS_peak_ and PB for HIS vs. control treatments (atorvastatin vs. control, adjusted *P* < 0.001; rosuvastatin vs. control, adjusted *P* < 0.001), indicating the relationship between ΔPSS_peak_ and PB differed between control and atorvastatin/rosuvastatin treatments. Notably, ΔPSS_peak_ increased when PB was above ∼50% in controls but was unchanged with either atorvastatin or rosuvastatin. A similar interaction effect occurred between baseline PA and treatment group, where PSS_peak_ increased when PA was above ∼8.0 mm^2^ in controls, and not with atorvastatin/rosuvastatin (*Figure 2C* and *D*).

To examine whether the relationship between PSS and PB/PA or treatment was due to differences in patient demographics, we undertook multivariable analyses of potential clinically-important confounding factors such as age, gender, hypertension, smoking status, diabetes, family history of CAD, and prior statin use. Despite differences in these parameters between groups, the interaction effect between atorvastatin/rosuvastatin treatment and PB remained (*Table 1*).

**Table 1:** Multivariable analysis to assess interaction between baseline plaque burden and treatment group on ΔPSS_peak_

Fixed-effect parameter	Estimate	Standard error	*P*-Value
Interaction: atorvastatin × baseline plaque burden	−1.37	0.23	**<0.0001**
Interaction: rosuvastatin × baseline plaque burden	−1.01	0.19	**<0.0001**
Age, as continuous variable	–	–	0.47
Gender, female vs. male	–	–	0.78
Hypertension	–	–	0.64
Current smoker	–	–	0.065
Diabetes	–	–	0.16
Family history of CAD	–	–	0.70
Prior statin use	–	–	0.25

CAD, coronary artery disease; PSS, plaque structural stress.

Bold text indicates statistical significance (i.e. p < 0.05).

We also examined the relationship between changes in PSS and changes in serum lipids. The effects of systemic lipid lowering on individual lesion PSS are not predictable, as PSS_peak_ varies markedly between frames^17^ and is highly localized to specific plaque regions related to both architecture and geometry, while PSS_mean_ averages values around the lumen circumference (*Figure 1D*). Changes in PSS_peak_ and PSS_mean_ were only weakly (and negatively) correlated with LDL changes in individual patients (and not correlated with changes in HDL) (Supplementary material online, *Figure S2*), suggesting that LDL reduction alone is not associated with reduced PSS.

### Effects of changes in plaque composition and geometry

**Figure 3:**
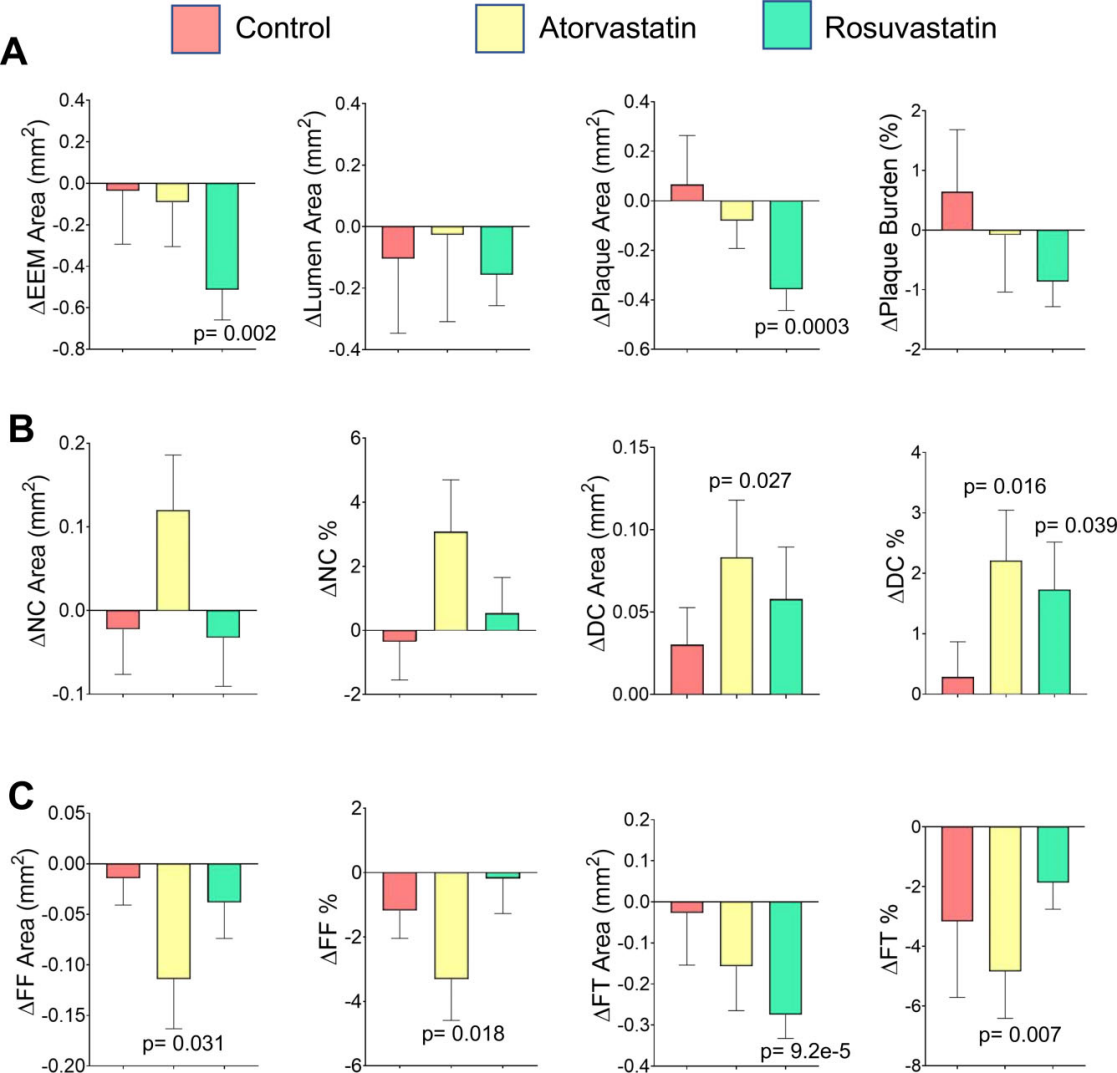
Changes in plaque geometric parameters or plaque constituents after control or high-intensity statin treatment. Change in (*A*) plaque geometric parameters including external elastic membrane area, plaque area and plaque burden, (*B*) necrotic core area and % or dense calcium area and %, and (*C*) fibrofatty area and % or fibrous tissue area and % in control or high-intensity statin-treated patients. Data are mean (standard error) between baseline and follow-up using mixed-effects models.

We next examined whether changes in peak and mean PSS with treatment were associated with changes in plaque geometric and compositional parameters. Control patients showed no significant change in any plaque characteristic. Atorvastatin-treated patients had reduced FF area and %, and FT area, and increased DC area and %. Rosuvastatin-treated patients showed decreased EEM, plaque, and FT areas, and increased DC % (*Figure 3*). However, ΔPSS_peak_ was only weakly correlated with Δlumen area, ΔPA or ΔPB in all plaques, although more strongly correlated with Δlumen aspect ratio, a measure of lumen ‘roundness’; in contrast, ΔPSS_mean_ was positively correlated with increasing lumen area and decreasing PA or PB, but not Δlumen aspect ratio (Supplementary material online, *Table S5*). Both ΔPSS_peak_ and ΔPSS_mean_ also correlated poorly with changes in NC, FF or FT areas or percentage, suggesting that effect of HIS on PSS_peak_ is not due to different effects on plaque composition alone. ΔPSS_peak_ was poorly correlated with calcification, although ΔPSS_mean_ was more strongly correlated with ΔDC area, ΔDC maximum and total arcs.

**Figure 4:**
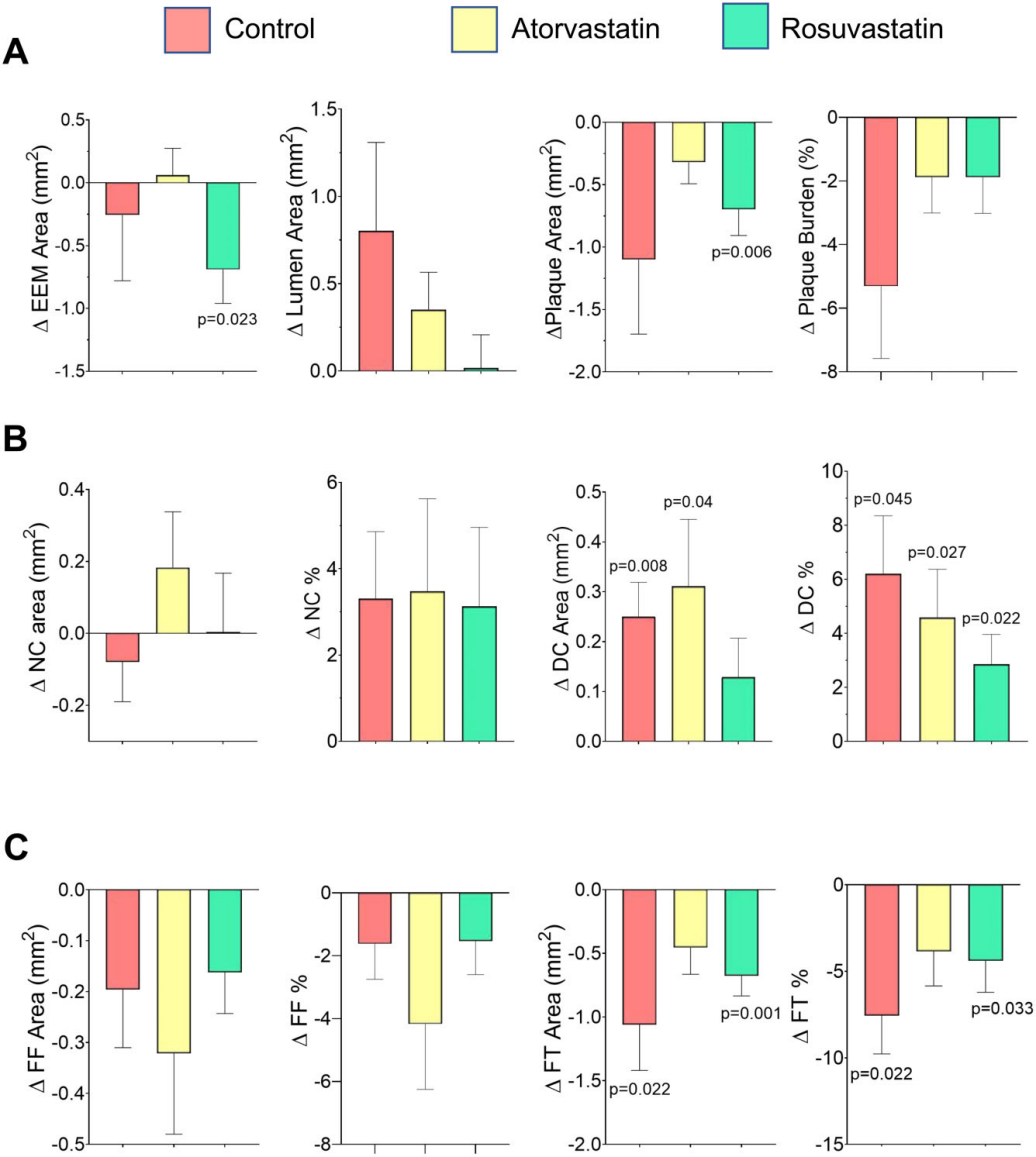
Changes in plaque geometric parameters or constituents in advanced lesions (PB > 60%) after control or high-intensity statin treatment. Changes in anatomical parameters including external elastic membrane area, plaque area and plaque burden (*A*), necrotic core area and percentage and dense calcium area and percentage (*B*), and fibrofatty area and percentage and fibrous tissue area, and percentage (*C*) in control patients or after high-intensity statin treatment. Data are mean (standard error) using mixed-effects models, *n* = 1112 frames, total 30 patients.

We further examined whether changes in PA or components explain PSS_peak_ differences in PB > 60% lesions between control and HIS treatments. Although changes in PA, burden or specific component parameters were significantly different between baseline and follow-up in control or atorvastatin/rosuvastatin patients, the direction of changes was similar in all groups (*Figure 4*). This indicates that different effects on plaque or component areas alone cannot explain why PSS_peak_ does not rise in PB >60% lesions with HIS treatment.

### Effects of changes in lumen geometry

**Figure 5:**
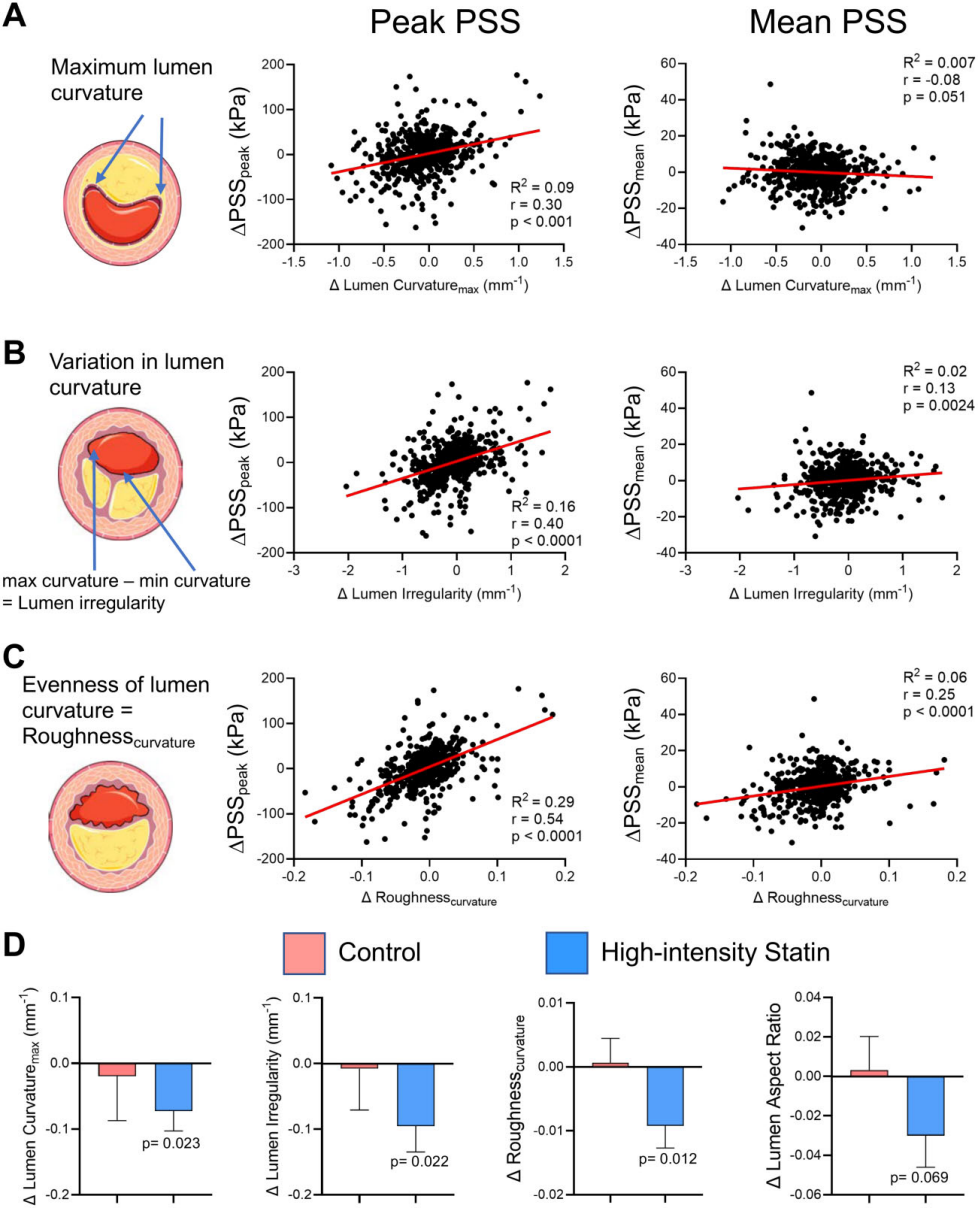
Lumen parameter analysis in plaques with baseline plaque burden >60%. Linear regression correlation curves for change in peak and mean plaque structural stress, with change in (*A*) maximum lumen curvature, (*B*) lumen irregularity, and (*C*) lumen roughness, in all plaques with baseline plaque burden >60%. (*D*) Change in lumen curvature, irregularity, roughness, and lumen aspect ratio in control patients or after high-intensity statin treatment. Data are mean (standard error) by plaque-level mixed effect models, total frames = 1112, plaques = 42.

Our data suggest that changes in PSS_peak_ reflect more localized changes in lumen and plaque geometry and plaque architecture, particularly at or close to the lumen/plaque interface. We therefore explored the effect of lumen curvature, lumen irregularity, and lumen roughness on PSS in PB >60% lesions, and their changes associated with treatment. Lumen curvature, irregularity, and roughness were all strongly positively correlated with ΔPSS_peak_ but poorly with ΔPSS_mean_ in PB >60% lesions (*Figure 5A–C*). As regression slopes of these lumen parameters with ΔPSS_peak_ were similar (*P* > 0.05) in atorvastatin and rosuvastatin groups (Supplementary material online, *Figure S3*), we examined changes in these parameters in a combined HIS treatment group vs. controls. HIS were associated with a significant reduction in lumen curvature, irregularity and lumen roughness, an effect not seen in controls (*Figure 5D*), with similar findings 4 mm proximal/distal to the minimal luminal area (MLA), a region highly correlated with MACE^17^ (Supplementary material online, *Table S6*).

### Effects of combinations of factors on ΔPSS_peak_ and ΔPSS_mean_

**Figure 6:**
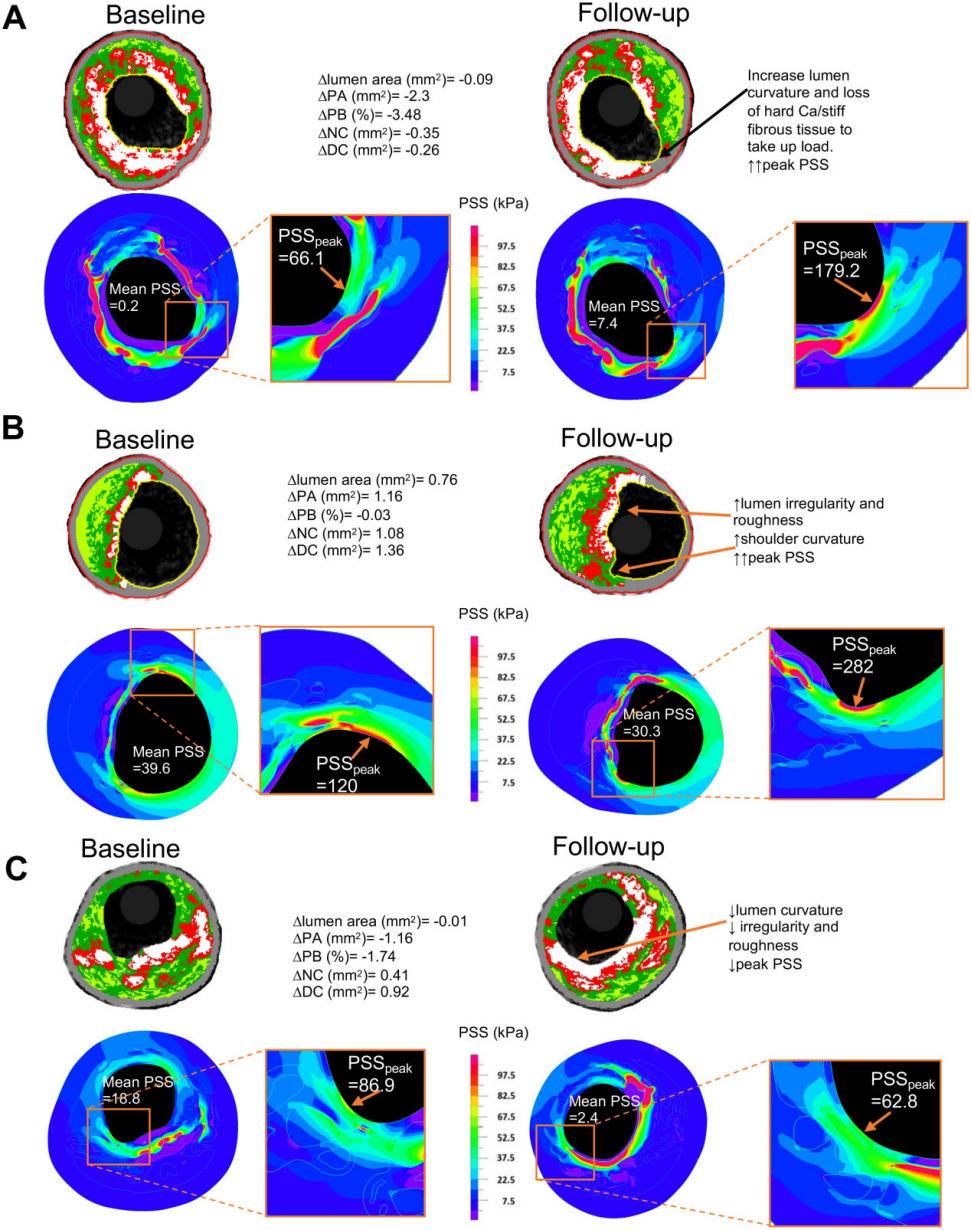
Examples of changes in plaque structural stress due to changes in lumen curvature, irregularity, roughness, and plaque architecture. (*A*) Increased PSS_peak_ due to changes in curvature and calcification/fibrous tissue arrangement. (*B*) Marked increase in PSS_peak_ due to increase in lumen curvature, irregularity and roughness. (*C*) Reduced PSS_peak_ due to changes in curvature, irregularity/roughness, and more confluent calcification. DC, dense calcium; NC, necrotic core; PA, plaque area; PB, plaque burden; PSS_peak_, peak plaque structural stress.

While ΔPSS_mean_ was mostly determined by anatomical factors and circumferential calcification, and ΔPSS_peak_ by localized luminal features, these parameters may all change together. Indeed, large increases or decreases in ΔPSS_mean_ and ΔPSS_peak_ occurred when changes in multiple features coincided across a range of PB (*Figure 6*). For example, increased PSS_peak_ was associated with increased lumen curvature and loss of ‘shielding’ calcification (*Figure 6A*) and increased lumen irregularity, roughness, and shoulder curvature (*Figure 6B*), while decreased PSS_peak_ was associated with reduced lumen irregularity, roughness, and curvature and increased confluence of superficial calcification (*Figure 6C*).

## Discussion

We undertook an observational study of three longitudinal trials of standard medical or HIS treatment, examining changes in PSS, plaque and lumen geometry and composition. We show that: (i) PSS_peak_ increased markedly in advanced lesions with standard medical but not HIS treatment; (ii) changes in PSS_peak_ were associated with both treatment and PB; (iii) changes in plaque and lumen area or plaque composition alone do not explain potential protective effects of HIS on ΔPSS_peak_; (iv) ΔPSS_peak_ is also affected by localized changes in plaque and lumen geometry, including lumen curvature, irregularity and roughness, while ΔPSS_mean_ is associated with changes in lumen and PA and circumferential calcification; and (v) HIS treatment is associated with remodelling lumen and plaque shape and architecture.

Previous landmark trials found that rosuvastatin 40 mg or atorvastatin 80 mg for 24 m reduce PAV by only ∼1%.^7^^,^^24^ Similarly, although differences exist between individual VH-IVUS studies, two meta-analyses showed only small or no change in FT or NC areas after statin treatment.^10^^,^^11^ Consistent with these meta-analyses, HIS treatment was not associated with reduced PB, and patients receiving standard medical or HIS treatment showed similar changes in NC and FF tissue area or %. Together, these findings suggest that small reductions in atheroma volume or these plaque components may not fully explain the ability of HIS to reduce MACE compared to standard therapy. In contrast, PSS_peak_ increased significantly in PB >60% lesions with standard treatment but not HIS, an action predicted to stabilize these higher-risk plaques.

Prospective natural history VH-IVUS studies showed that PB ≥70%, MLA <4 mm, and VH-IVUS-defined thin cap atheromas were associated with future MACE^4^^,^^5^^,^^23^; however, the overall low event rates suggest that factors additional to plaque size, stenosis and composition determine rupture. PSS measurements integrate effects of plaque anatomy and composition with physical forces, and inclusion of PSS measurements improve future MACE prediction,^16^^,^^17^ especially in higher-risk regions. We therefore identified the parameters associated with changes in both mean and peak PSS. ΔPSS_mean_ correlated with changes in lumen and PA and PB, consistent with Laplace’s law where mean wall stress increases with intracavity pressure or increasing vessel radius (when plaques regress) or vice versa (when plaques progress). ΔPSS_mean_ also correlated with circumferential calcification, which can act as either stress amplifiers or lumen cap protectors depending on size, orientation, and confluence. Larger calcification plates (>1 mm) may stabilize plaques by shielding from luminal stress,^25^ and atorvastatin/rosuvastatin increased DC percentage, a feature shown consistently in statin trials.^10^^,^^11^ Current algorithms for total DC area, arc or contour lack the ability to detect subtle changes in plaque microstructure. In contrast, PSS estimation at higher-risk plaque regions represents an objective method to quantify microstructural differences.

PSS_peak_ is normally located in the superficial 0.2 mm of the lesion,^14^ and at maximum curvature at the plaque shoulder, a frequent site of rupture.^26^ ΔPSS_peak_ also correlated with changes in lumen curvature, irregularity and roughness, which measure both large and small lumen/plaque irregularities. These parameters were reduced in PB >60% lesions of patients receiving HIS, potentially explaining the absence of a PSS_peak_ rise seen with HIS treatment. Plaque/luminal irregularity, defined as a rough lumen surface along the direction of blood flow, is a strong predictor of plaque instability,^22^ while repetitive silent rupture or erosion may generate new areas of acute angulation and roughness.

The mechanisms by which HIS might reduce or prevent a rise in PSS_peak_ are not known, and may be multiple. We found a weak negative correlation between ΔPSS and ΔLDL, and the interaction effect of baseline PB and treatment group on PSS was independent of prior statin use, suggesting that LDL lowering alone does not reduce PSS. However, statins also increase nitric oxide (NO) bioavailability, which improves endothelial cell function,^27^ suppresses coagulation by inhibiting platelet adhesion and aggregation,^28^ and blocks endothelial cell apoptosis.^29^ Improved endothelial function and better reorganization of luminal thrombus may also smooth the plaque surface.

Our study has several limitations. First, trials were performed in two different centres at different times. However, the same VH-IVUS and PSS platforms were used throughout, so comparable images and PSS calculations were obtained. Second, patient demographics and plaque characteristics were not propensity-matched, including prior statin use, and PB and PA differed between trials. However, we analysed changes in PSS and plaque and lumen features between baseline and follow-up in all patients where each patient acts as their own control, LDL reduction was not correlated with decreased PSS, PSS was examined across a full range of PB (reflecting real-world patient presentation), and our main findings remained after multivariable regression analysis for confounding factors. Third, VH-IVUS has well-documented limitations to identify and measure plaque components, including fibrous cap thickness that correlates negatively with PSS_peak_. However, our frame-based analysis was verified in 2-mm segments, parameters that segregate different treatments (PA, DC percentage and arc, lumen curvature, irregularity, and roughness) are all within VH-IVUS resolution. Lastly, since these studies did not examine MACE, so that our findings are hypothesis generating and require further work to determine how HIS can remodel the lumen/plaque interface.

## Conclusion

We find that changes in PSS_peak_ are associated with complex interactions between plaque architecture, lumen geometry, baseline disease severity, and treatment. PSS increased over time in advanced lesions in patients receiving standard medical treatment, but not with HIS, associated with remodelling artery geometry and plaque architecture. Smoothing plaques and reducing lumen curvature represent novel mechanisms whereby HIS may protect against plaque rupture.

## Lead author biography

**Figure:**
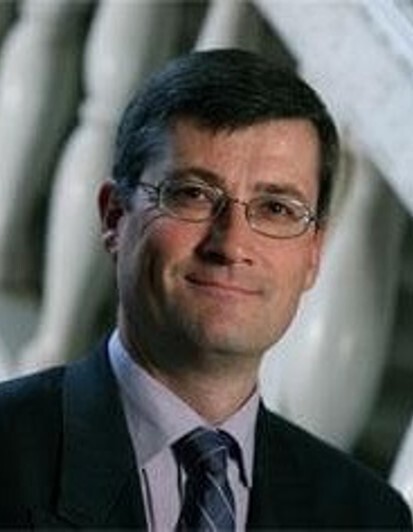


Professor Martin R. Bennett holds the British Heart Foundation (BHF) Chair of Cardiovascular Sciences at the University of Cambridge, directs the BHF Cambridge Centre for Cardiovascular Research Excellence, and holds Honorary Consultant Cardiologist positions at Cambridge University and Royal Papworth Hospitals. His clinical research programme combines clinical medicine, coronary imaging, and engineering for vulnerable plaque detection.

## Supplementary material

Supplementary material is available at *European Heart Journal Open* online.

## Funding

British Heart Foundation (BHF) (grants FS/19/66/34658, PG/16/24/32090, RG71070, and RG84554); the National Institute of Health Research Cambridge Biomedical Research Centre; and the BHF Centre for Research Excellence.

**Conflict of interest:** LR declares grants from Abbott, Biotronik, Boston Scientific, Heartflow, Infraredx, Sanofi, and Regeneron and consulting fees from Abbott, Amgen, AstraZeneca, Canon, Medtronic, Occlutech, Sanofi, and Vifor.

## Data availability

The data underlying this article will be shared on reasonable request to the corresponding author.

## Supplementary Material

oeab039_Supplementary_Data
