# Supplementary material for: High-intensity statin treatment is associated with reduced plaque structural stress and remodelling of artery geometry and plaque architecture
Source: Eur Heart J Open. 2021 Nov 17;1(3):oeab039. doi: 10.1093/ehjopen/oeab039 (PMC9242039; doi:10.1093/ehjopen/oeab039)
Supplement: oeab039_Supplementary_Data [file oeab039_supplementary_data.pdf]

SUPPLEMENTAL MATERIAL

Supplemental Methods

Biomechanical modeling of plaque structural stress

Plaque geometry was constructed from VH-IVUS images using an in-house developed MATLAB code (proprietary code, MATLAB R2020a, MathWorks, Inc, Natick, Massachusetts, USA). Each VH-IVUS frame was segmented into its individual components, and a 5% circumferential shrinkage applied to generate a zero-pressure condition as *in vivo* data were recorded during diastole. A 65μm layer of fibrous tissue was introduced during mesh generation to account for the limited axial resolution of VH-IVUS to detect a fibrous cap. The vessel wall and all plaque components were assumed to be hyper-elastic, non-linear, isotropic, incompressible, and piecewise homogeneous. The modified Mooney-Rivlin model was used to describe the material property of each component:

$$W = c_1(\bar{I}_1 - 3) + D_1\left[e^{D_2(\bar{I}_1 - 3)} - 1\right] + \kappa(J - 1)$$

where $\bar{I}_1 = J^{-2/3}I_1$ with I_1 being the first strain invariant of the unimodular component of the left Cauchy-Green deformation tensor. $J = det(\boldsymbol{F})$ and $\boldsymbol{F}$ is the deformation gradient. κ is the Lagrangian multiplier for the incompressibility. c_1, D_1 and D_2 are material parameters derived from direct material testing results. In this study, the following values were used: arterial wall, c_1=0.14 kPa, D_1=3.83 kPa, D_2=18.80 kPa; fibrous tissue, c_1=0.19 kPa, D_1=5.77 kPa, D_2=18.22 kPa; necrotic core, c_1=0.05 kPa, D_1=4.89 kPa, D_2=5.43 kPa and dense calcification, c_1=1.15x10^5 kPa, D_1=7.67x10^4 kPa, D_2=2.84x10^{-8} kPa.[1,2] The motion of each atherosclerotic component is governed by kinetic equations as:

$$\rho v_{i,tt} = \sigma_{ij,j} \; (i, j = 1, 2)$$

where $[v_i]$ and $[\sigma_{ij}]$ are the displacement vector and stress tensor, respectively, ρ is the density of each component, and t is time.

The entire plaque geometric model was meshed using 9-node quadrilaterals, generating approximately 10,000 elements and 40,000 nodes per model. Displacement and strain were assumed to be large. There was no relative movement at the interface of atherosclerotic components and the relative energy tolerance was set to be 0.005. Two adjacent points located on the outer wall were fixed to prevent rigid body displacement. Maximum principal stress was used to characterize the mechanical loading within the plaque structure (PSS) in the peri-luminal region (0.2mm maximum depth from the luminal contour). Mean PSS was calculated as the mean value of PSS experienced by all the luminal nodes. Dynamic loading conditions were standardized to 120/70mmHg. Pressure at the outer boundary was set to zero. All simulations were performed using ADINA 9.5 (ADINA R&D, Inc., USA) software.

Additional measures (*Figure S1*):

- **Lumen aspect ratio** = maximum diameter of ellipse (or lumen major axis)/minimum diameter of ellipse (or lumen minor axis), i.e., lower (improved) aspect ratio describes a rounder lumen, and a value of 1 indicates a perfectly circular lumen.
- **Lumen curvature:**[3,4] curvature at point a (in ***Figure S1***) was computed using the radius (as r_a) of the circle determined by point a and two adjacent points (bottom right figure) on both sides, i.e. curvature = $1/r_a$. Curvature value was computed for all points in the lumen, and the maximum lumen curvature value (Lumen Curvature_{max}) is used in data analysis. The minimum lumen curvature value (Lumen Curvature_{min}) is also computed for lumen irregularity calculation

- **Lumen irregularity**[5] = Lumen $Curvature_{max}$ – Lumen $Curvature_{min}$
- **Lumen roughness:** reflects the lumen surface evenness in respect to curvature, and calculated using the following formula, with smaller values representing more round or even surface and a perfect round lumen shape will have roughness being 1. Method adapted from.[6]

$$Roughness_{Curvature} = \sqrt{\frac{1}{2\pi r} \sum \left(\frac{r}{r_a}\right)^2 \Delta l}$$

(r is the radius of the circle best fitting the lumen contour; r_a is defined as above in lumen curvature calculation; and Δl is the length between point a and one adjacent point.)

Assessment of analyst variability

The reproducibility of matching between baseline and follow-up VH-IVUS frames by 2 analysts was examined in 6 vessels that had both baseline (n= 573 frames) and follow-up (n= 623 frames). The 2 analysts reviewed the VH-IVUS data and separately identified the location of follow-up frames in the 2mm segments defined in the baseline frames. To report the intra-observer variability the 1st analyst performed the analysis twice. The κ test of concordance was used to assess agreement. A good overall agreement was noted for the estimation of the two analysts with the intra-observer variability being 0.733 and the inter-observer variability being 0.701. The reproducibility of lumen curvature, irregularity, and roughness assessment was examined on 2 randomly selected vessels (77 frames) by testing the intraclass correlation coefficient (ICC); this achieved good to excellent absolute agreement: lumen curvature, ICC= 0.787; lumen irregularity, ICC = 0.72; lumen roughness, ICC= 0.712.

Statistical analysis of patient demographics

Continuous variables are presented as mean ± standard deviation or median (interquartile range) and discrete variables as absolute numbers (percentage). Normality tests were performed for all variables using quantile-quantile plots, and Kolmogorox-Smirnov/Shapiro-Wilks test where appropriate. Student's t-test or one-way ANOVA were used for normally distributed continuous variables. Non-normally distributed variables were analyzed using Mann-Whitney U test or Kruskal-Wallis test for independent samples, and Wilcoxon signed-rank test for paired samples. Chi-square test (χ^2) or Fisher's exact test was used for discrete variables where appropriate. We identified a number of potential clinically important confounding factors (age as continuous variable, gender, hypertension, smoking status, diabetes, family history of coronary artery disease, and prior statin use), and these were added in the multivariable model as fixed effects to examine our main study finding.

Supplemental Tables

Table S1. Trial groups, inclusion and exclusion criteria

	Control	Atorvastatin	Rosuvastatin
Treatment	Aspirin, low-intensity statin, β-blocker	Atorvastatin 80mg	Rosuvastatin 40mg
Trial registration	NCT01230892	NCT00576576	NCT00962416
Patient number	n= 18	n= 20	n= 22
Follow-up period	12 months	6 months	13 months
**Inclusion criteria**			
Presentation	Stable angina or NSTEMI	Stable angina or ACS	STEMI
Age	21 to 79 years	≥ 18 years	18 to 89 years
Lesion	Moderate lesions requiring physiologic assessment<br>On stable medical therapy	Moderate lesions requiring invasive physiologic evaluation	2 major proximal arteries suitable for intracoronary imaging
**Exclusion criteria**			
Hemodynamic	STEMI<br>Cardiogenic shock	STEMI, cardiogenic shock, hemodynamic instability	Acute MI due to stent thrombosis<br>Mechanical complication of acute MI
Lesion specific	Lesions requiring revascularization<br>LM>50% stenosis<br>Lesion beyond 60mm<br>Significant visual collaterals	Lesions requiring PCI or CABG<br>LM >50% stenosis<br>Lesion beyond 60mm<br>Visual collaterals	Lesions requiring treatment (stenosis>50%) in 2 major proximal arteries<br>Infarct lesion at site of a previously implanted stent
Other cardiac history	CABG<br>Severe valvular heart disease<br>EF<30%	CABG<br>severe valvular heart disease	-
Treatment	Contraindication to β-blockers, CCBs or extended-release nitrate therapy within last 48 hours	On maximum dose of statin<br>On statin with an LDL≤130mg/dl	Known intolerance to aspirin, clopidogrel, heparin, stainless steel, biolimus or contrast material
Other comorbidities	Creatinine>1.5mg/dL, renal impairment<br>Liver impairment	Creatinine>1.5mg/dL<br>Liver disease<br>Uncontrolled diabetes<br>Uncontrolled hypertension	Renal failure<br>Planned surgery within 6 months of PCI<br>Life expectancy <1 year
Pregnancy	-	Pregnancy or planned pregnancy	Female of childbearing potential
Coagulopathy	Hematologic disease	INR>1.8<br>Hematologic disease	Bleeding diathesis/known coagulopathy<br>Use of warfarin
Other trial	-	-	Currently participating in another trial before reaching first endpoint

ACS, acute coronary syndrome; CABG, coronary artery bypass graft; CCB, calcium channel blocker; EF, ejection fraction; INR, international normalized ratio; LDL, low-density lipoproteins; LM, left main stem artery; MACE, major adverse cardiac events; MI, myocardial infarction; NSTEMI, non-ST elevation myocardial infarction; PCI, percutaneous coronary intervention; STEMI, ST-segment elevation myocardial infarction

Table S2. Patient demographic and clinical characteristics

	Control (C) n=18	Atorvastatin (A) n=20	Rosuvastatin (R) n=22	P value		
				C vs. A	C vs. R	A vs. R
Age, years (mean ± SD)	51.0 ±10.2	55.9 ± 12.5	57.6 ± 9.7	0.36	0.14	0.855
Male, n (%)	8 (44.4)	13 (65)	20 (90.9)	0.203	**0.002**	0.062
BMI, kg/m^2 (mean ± SD)	29.2 ± 5.8	31.9 ± 5.9	27.2 ± 3.8	0.259	0.451	**0.014**
Hypertension, n (%)	12 (66.7)	14 (70)	11 (50)	0.825	0.289	0.187
Current smoking, n (%)	1 (5.6)	5 (25)	11 (50)	0.184	**0.004**	0.096
Diabetes, n (%)	3 (16.7)	6 (30)	2 (9.1)	0.454	0.642	0.123
Hypercholesterolemia n (%)	12 (66.7)	17 (85)	8 (36.4)	0.26	0.057	**0.002**
Family history of CAD, n (%)	8 (44.4)	7 (35)	5 (22.7)	0.552	0.145	0.379
Previous MI, n (%)	4 (22.2)	2 (10)	1 (4.5)	0.395	0.155	0.598
Previous PCI	5 (27.8)	4 (20)	1 (4.5)	0.709	0.073	0.174
**Presentation**						
Stable angina, n (%)	13 (72.2)	13 (65)	0	0.632	-	-
ACS, n (%)	5 (27.8)	7 (35)	0	0.632	-	-
STEMI, n (%)	0 (0)	0 (0)	22 (100)	-	-	-
**Prior Medications**						
Statin, n (%)	12 (66.7)	4 (20)	1 (4.5)	**0.008**	**<0.001**	0.174
β-blockers, n (%)	7 (38.9)	8 (40)	2 (9.1)	0.944	0.053	**0.03**
Aspirin, n (%)	13 (72.2)	13 (65)	1 (4.5)	0.632	**<0.001**	**<0.001**
Antiplatelet, n (%)	5 (27.8)	3 (15)	0 (0)	0.438	**0.013**	0.099
CCB, n (%)	5 (27.8)	2 (10)	1 (4.5)	0.222	0.073	0.598
Nitrate, n (%)	13 (72.2)	4 (20)	0 (0)	**0.003**	**<0.001**	**0.043**
ACE inhibitor or ARB, n (%)	5 (27.8)	10 (50)	5 (22.7)	0.162	0.714	0.065
**Lipid levels**						
Change in LDL, mg/dL (mean ± SD)	**17.2 ± 35.8***	**-47.5 ± 30.5†**	**-29.8 ± 38.2‡**	**<0.001**	**<0.001**	0.256
Change in HDL, mg/dL (mean ± SD)	0.4 ± 10.8§	1.8 ± 8.5‖	**5.0 ± 8.4¶**	0.869	0.285	0.551
**Blood pressure**						
Change in mean arterial pressure, mmHg (mean ± SD)	-2.6 ± 15.5	0.1 ± 16.3	-2.7 ± 13.3	0.852	0.999	0.853

ACE, angiotensin converting enzyme; ACS, acute coronary syndrome; ARB, angiotensin receptor blocker; BMI, body mass index; CAD, coronary artery disease; CCB, calcium channel blocker; HDL, high-density lipoprotein; LDL, low-density lipoprotein; MI, myocardial infarction; NSTEMI, non-ST segment elevation myocardial infarction; SD, standard deviation; STEMI, ST-segment elevation myocardial infarction; UA, unstable angina.

*p=0.031; † p<0.001; ‡ p=0.003; § p=0.877; ‖ p=0.308; ¶ p=0.014.

Table S3. Baseline VH-IVUS characteristics

Characteristics, mean ± SE	**Control (C) frame n=766 patient=18**	**Atorvastatin (A) frame n=1218 patient=20**	**Rosuvastatin (R) frame n=1690 patient=22**	**P value**		
				**C vs. A**	**C vs. R**	**R vs. A**
EEM area, mm^2	16.71 ± 0.20	16.29 ± 0.14	14.18 ± 0.11	0.902	**0.028**	**0.033**
Lumen area, mm^2	10.21 ± 0.15	9.11 ± 0.11	7.16 ± 0.06	0.355	**0.001**	**0.01**
Plaque area, mm^2	6.50 ± 0.11	7.18 ± 0.08	7.01 ± 0.07	0.304	0.663	0.515
Plaque burden (%)	39.6 ± 0.49	44.4 ± 0.38	48.5 ± 0.29	0.122	**0.003**	0.156
NC%	17.7 ± 0.45	18.5 ± 0.34	20.2 ± 0.32	0.823	0.327	0.421
NC area, mm^2	0.70 ± 0.03	0.80 ± 0.02	1.00 ± 0.02	0.578	0.16	0.357
DC%	6.31 ± 0.33	7.40 ± 0.26	8.00 ± 0.23	0.268	0.164	0.761
DC area, mm^2	0.23 ± 0.01	0.33 ± 0.01	0.41 ± 0.01	0.107	0.052	0.532
FT%	65.9 ± 0.72	64.7 ± 0.48	57.9 ± 0.49	0.395	**0.004**	**0.021**
FT area, mm^2	1.92 ± 0.06	2.39 ± 0.05	2.21 ± 0.03	0.258	0.592	0.441
FF%	6.90 ± 0.23	8.75 ± 0.23	9.34 ± 0.23	0.093	0.198	0.667
FF area, mm^2	0.22 ± 0.01	0.39 ± 0.01	0.33 ± 0.01	**0.034**	0.25	0.167

Data are mean (SE)

DC, dense calcification; EEM, external elastic membrane; FF, fibrofatty tissue; FT, fibrous tissue; NC, necrotic core; SE, standard error; VH-IVUS, virtual histology intravascular ultrasound

Table S4. Segmental analysis on changes in peak and mean plaque structural stress with different statin regimes and baseline disease severity

	**Control**		**Atorvastatin**		**Rosuvastatin**	
**Overall**	Segment=237	P value	Segment=374	P value	Segment=445	P value
ΔPeak PSS, kPa (mean ± SE)	-8.6 ± 3.6	**0.03**	6.2 ± 5.9	0.306	-1.4 ± 1.8	0.446
ΔMean PSS, kPa (mean ± SE)	-1.1 ± 1.5	0.481	1.2 ± 1.2	0.34	-0.5 ± 0.6	0.399
**Baseline PB<40%**	Segment=141	P value	Segment=165	P value	Segment=94	P value
ΔPeak PSS, kPa (mean ± SE)	-16.7 ± 5.0	**0.004**	9.1 ± 8.0	0.272	-2.9 ± 3.4	0.405
ΔMean PSS, kPa (mean ± SE)	-2.4 ± 1.8	0.2	1.7 ± 1.9	0.368	0.2 ± 0.8	0.82
**Baseline PB=40-60%**	Segment=71	P value	Segment=168	P value	Segment=269	P value
ΔPeak PSS, kPa (mean ± SE)	-2.3 ± 4.5	0.608	6.6 ± 5.2	0.224	-1.2 ± 2.0	0.562
ΔMean PSS, kPa (mean ± SE)	-0.7 ± 1.3	0.625	0.8 ± 1.1	0.466	-1.1 ± 0.6	0.076
**Baseline PB>60%**	Segment=25	P value	Segment=41	P value	Segment=82	P value
ΔPeak PSS, kPa (mean ± SE)	19.4 ± 6.1	0.058	-7.2 ± 7.1	0.412	-2.0 ± 5.7	0.735
ΔMean PSS, kPa (mean ± SE)	1.5 ± 3.4	0.681	-0.2 ± 1.9	0.936	-0.2 ± 1.4	0.88

PB, plaque burden; PSS, plaque structural stress; SE, standard error.

Table S5. Correlation between changes in peak and mean PSS and plaque geometric and compositional parameters

	**ΔPSS_{peak} (kPa)**			**ΔPSS_{mean} (kPa)**		
	Correlation coefficient (r)	R^2	p	Correlation coefficient (r)	R^2	p
ΔLumen area (mm^2)	0.297	0.088	**<0.0001**	0.584	0.34	**<0.0001**
ΔPlaque area (mm^2)	-0.16	0.026	**<0.0001**	-0.4	0.16	**<0.0001**
ΔPlaque burden (%)	-0.261	0.068	**<0.0001**	-0.6	0.36	**<0.0001**
ΔLumen aspect ratio	0.346	0.12	**<0.0001**	0.026	0.0007	0.11
ΔNC area (mm^2)	-0.024	0.0006	0.142	-0.16	0.026	**<0.0001**
ΔNC %	0.033	0.001	**0.046**	-0.064	0.004	**<0.0001**
ΔFF area (mm^2)	-0.071	0.005	**<0.0001**	-0.116	0.014	**<0.0001**
ΔFF %	-0.0046	2.1e-5	0.78	-0.051	0.003	**0.002**
ΔFT area (mm^2)	-0.151	0.023	**<0.0001**	-0.272	0.074	**<0.0001**
ΔFT %	-0.061	0.004	**0.0002**	-0.072	0.005	**<0.0001**
ΔDC area (mm^2)	-0.01	0.0001	0.52	-0.33	0.11	**<0.0001**
ΔDC %	0.05	0.0026	**0.0022**	-0.202	0.041	**<0.0001**
ΔMaximum arc of DC (°)	0.02	0.0004	0.21	-0.417	0.17	**<0.0001**
ΔTotal arc of DC (°)	-0.013	0.0002	0.44	-0.428	0.18	**<0.0001**

DC, dense calcium; FF, fibrofatty; FT, fibrous tissue; NC, necrotic core; PSS, plaque structural stress.

Table S6. Peri-MLA analysis on changes in lumen geometric features in plaques with baseline PB>60%

Characteristics<br>mean ± SE	Control		High-intensity statin	
	**frame =84**	**p**	**frame =412**	**p**
ΔCurvature$_{max}$ (mm^{-1})	-0.070 ± 0.090	0.464	-0.0773 ± 0.0378	0.0513
ΔIrregularity (mm^{-1})	-0.113 ± 0.0769	0.196	-0.139 ± 0.0544	**0.0174**
ΔRoughness$_{curvature}$	-0.00638 ± 0.00816	0.462	-0.0161 ± 0.00583	**0.0108**
ΔLumen aspect ratio	-0.010 ± 0.024	0.678	-0.059 ± 0.021	**0.01**

MLA, minimal luminal area; PB, plaque burden; SE, standard error.

Supplemental Figures

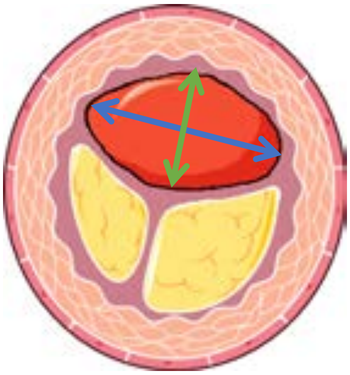

- Lumen aspect ratio = $\frac{\textit{lumen major axis}}{\textit{lumen minor axis}}$

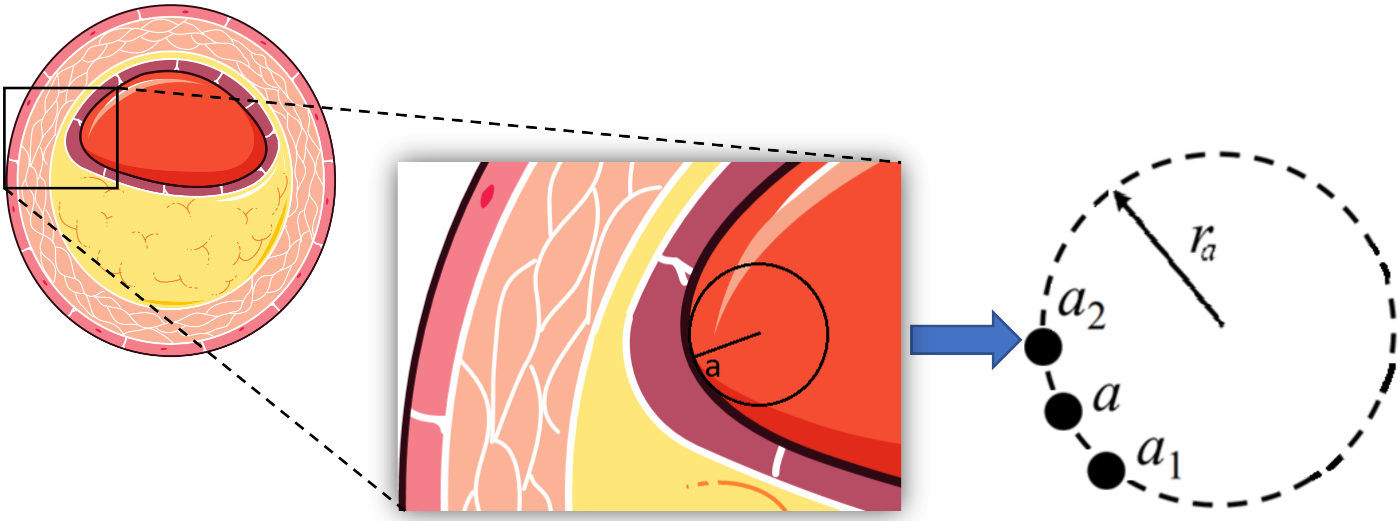

- Lumen curvature: curvature at point a was computed using the radius (as r_a) of the circle determined by point a and two adjacent points (a_1 and a_2), i.e. Lumen Curvature = $1/r_a$.

- Lumen Irregularity= Lumen Curvature_{max} – Lumen Curvature_{min}

- $$Roughness_{Curvature} = \sqrt{\frac{1}{2\pi r} \sum \left(\frac{r}{r_a}\right)^2 \Delta l}$$

  - Roughness as a measure of evenness of lumen curvature. r is the radius of the circle best fitting the lumen contour (i.e. lumen area $=\pi r^2$), Δl is the length between point a and one adjacent point

Figure S1. Definitions of lumen aspect ratio, curvature, irregularity, and roughness

Figure S2

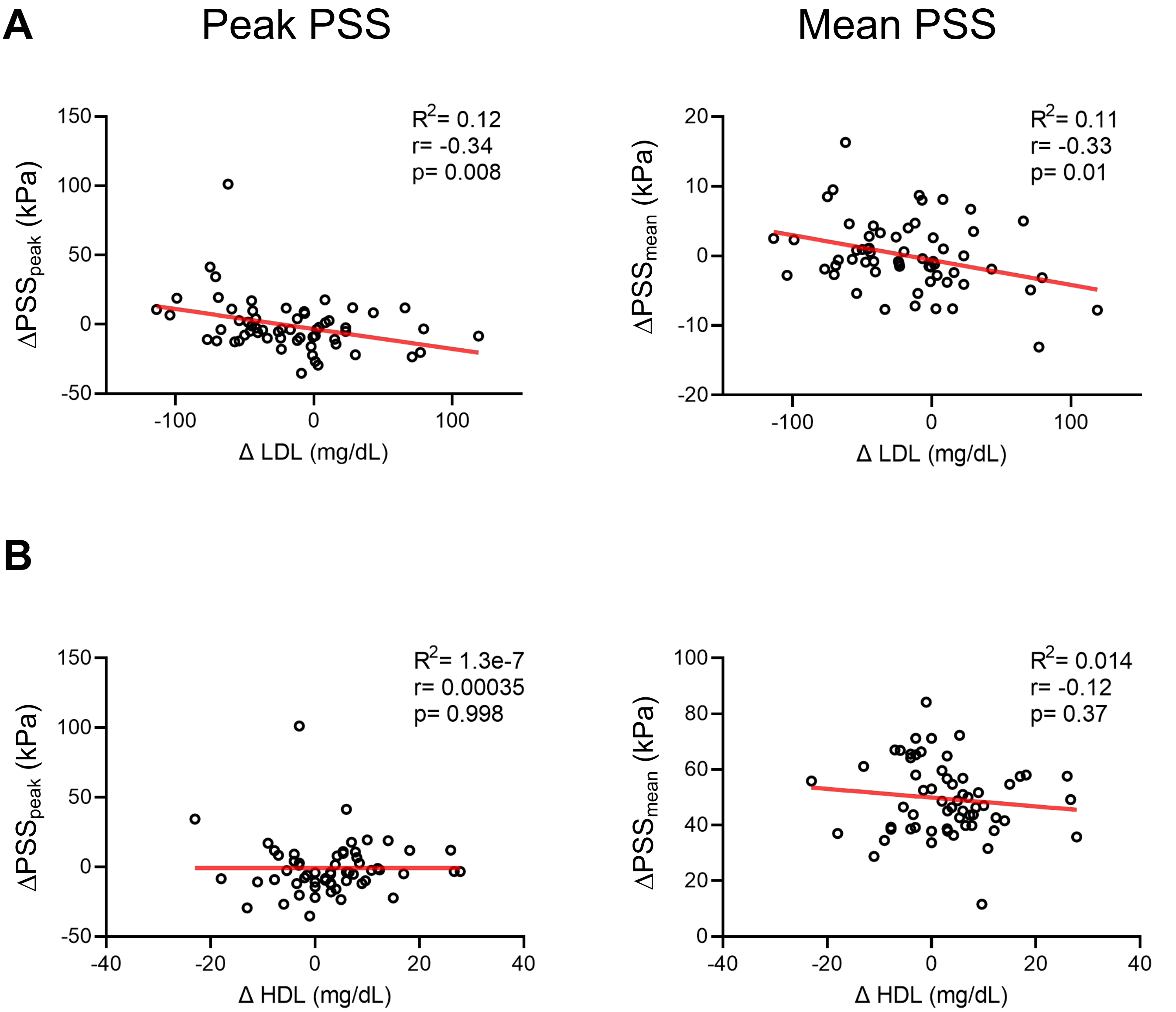

Figure S2. Association between change in PSS and change in lipid levels.
Linear correlation curves for change in peak (left) and mean PSS (right) with change in **(A**) LDL, **(B)** HDL. LDL or HDL changes are values for follow-up minus baseline, such that a higher negative value indicates a greater reduction from treatment. HDL= high-density lipoprotein; LDL= low-density lipoprotein; PSS = plaque structural stress.

Figure S3

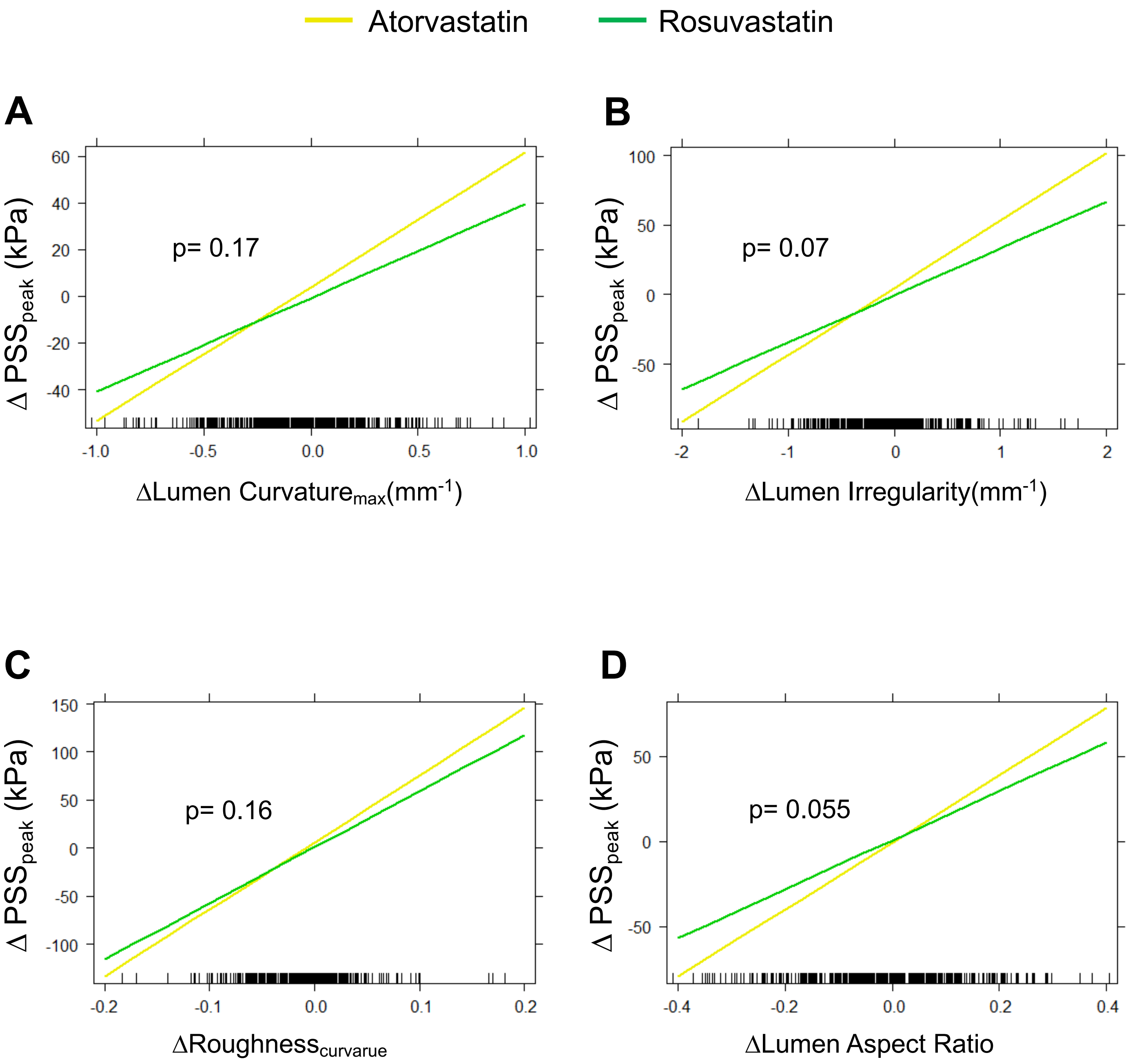

Figure S3. Correlation between changes in peak PSS and lumen parameters in atorvastatin and rosuvastatin groups in plaques with baseline PB>60%.
(A) maximum lumen curvature, **(B)** lumen irregularity, **(C)** lumen roughness **(D)** lumen aspect ratio. These regression slopes between the 2 high-intensity statin groups are similar ($p>0.05$).

Supplemental References

1. Ebenstein DM, Coughlin D, Chapman J, Li C, Pruitt LA. Nanomechanical properties of calcification, fibrous tissue, and hematoma from atherosclerotic plaques. *J Biomed Mater Res Part A* John Wiley & Sons, Ltd; 2009;**91A**:1028–1037.
2. Teng Z, Zhang Y, Huang Y, Feng J, Yuan J, Lu Q, Sutcliffe MPF, Brown AJ, Jing Z, Gillard JH. Material properties of components in human carotid atherosclerotic plaques: A uniaxial extension study. *Acta Biomater* Acta Materialia Inc.; 2014;**10**:5055–5063.
3. Teng Z, Sadat U, Li Z, Huang X, Zhu C, Young VE, Graves MJ, Gillard JH. Arterial Luminal Curvature and Fibrous-Cap Thickness Affect Critical Stress Conditions Within Atherosclerotic Plaque: An In Vivo MRI-Based 2D Finite-Element Study. *Ann Biomed Eng* 2010;**38**:3096–3101.
4. Akyildiz AC, Speelman L, Nieuwstadt HA, Brummelen H van, Virmani R, Lugt A van der, Steen AFW van der, Wentzel JJ, Gijsen FJH. The effects of plaque morphology and material properties on peak cap stress in human coronary arteries. *Comput Methods Biomech Biomed Engin* Taylor & Francis; 2016;**19**:771–779.
5. Teng Z, Sadat U, Ji G, Zhu C, Young VE, Graves MJ, Gillard JH. Lumen Irregularity Dominates the Relationship Between Mechanical Stress Condition, Fibrous-Cap Thickness, and Lumen Curvature in Carotid Atherosclerotic Plaque. *J Biomech Eng* 2011;**133**.
6. Teng Z, Degnan AJ, Sadat U, Wang F, Young VE, Graves MJ, Chen S, Gillard JH. Characterization of healing following atherosclerotic carotid plaque rupture in acutely symptomatic patients: an exploratory study using in vivo cardiovascular magnetic resonance. *J Cardiovasc Magn Reson* 2011;**13**:64.
